# Development of a stable liquid formulation of live attenuated influenza vaccine

**DOI:** 10.1016/j.vaccine.2016.04.074

**Published:** 2016-07-12

**Authors:** Jessica A. White, Marcus Estrada, E. Alexander Flood, Kutub Mahmood, Rajeev Dhere, Dexiang Chen

**Affiliations:** a PATH, Seattle, Washington, United States; b Serum Institute of India Pvt Ltd, Pune, MH, India

**Keywords:** Vaccine stabilization, Influenza vaccine

## Abstract

Vaccination is the most effective means of preventing influenza. However, the cost of producing annual seasonal influenza vaccines puts them out of reach for most developing countries. While live attenuated influenza vaccines are among the most efficacious and can be manufactured at low cost, they may require lyophilization to be stable enough for developing-country use, which adds a significant cost burden. The development of a liquid live attenuated seasonal influenza vaccine that is stable for around a year—the duration of an annual influenza season—would significantly improve not only the production output but also the use and accessibility of influenza vaccines in low-resource settings.

In this study, potential stabilizing excipients were screened and optimized using the least stable influenza vaccine strain presently known, H1N1 (A/California/07/2009), as a model. The stability-conferring properties of the lead formulations were also tested with a Type B strain of influenza virus (B/Brisbane/60/2008). Stability was also evaluated with higher titers of influenza virus and exposure to agitation and freeze–thaw stresses to further confirm the stability of the lead formulations. Through this process, we identified a liquid formulation consisting of sucrose phosphate glutamate buffer with 1% arginine and 0.5% recombinant human serum albumin that provided storage stability of one year at 2–8 °C for the influenza A and B strains tested.

## Highlights

• Multiple excipients were screened for their ability to stabilize influenza vaccine.
• Identified a liquid formulation with storage stability for one year at 2–8 °C.
• A stable liquid formulation may lead to wider use of influenza vaccination.

## 1 Introduction

Seasonal influenza affects millions of people each year, causing morbidity, mortality, and economic loss [1]. Vaccination is essential to prevent influenza virus infections [2] and is especially important to prevent influenza pandemics [3].

Influenza strains are characterized by surface glycoproteins and are classified into three types: A, B, and C strains. Because only strains A and B cause respiratory disease, vaccines target these subtypes. Within each subtype, influenza viruses are further categorized based on antigenic determinants in their surface glycoproteins hemagglutinin (HA) and neuraminidase (NA) [2], [4].

Influenza viruses undergo constant antigenic drift and antigenic shift. Point mutations in viral RNA during viral replication cause antigenic drift [4]. Antigenic shift occurs when two influenza strains exchange genome segments in a process called reassortment, resulting in a new virus [3]. Recommended influenza vaccines change each year due to frequent, rapid changes in antigenic determinants in circulating strains [5]. Immune responses generated by vaccination are directed against the HA and NA proteins [4].

Two main types of vaccines are licensed to prevent seasonal influenza. These are inactivated influenza vaccines (IIVs) and live attenuated, cold-adapted influenza virus vaccines (LAIVs). IIVs are licensed for intramuscular delivery to anyone six months of age or older and exist as inactivated whole virus particles or as split virion and recombinant subunit vaccines [2], [6], [7], [8], [9], [10]. Although millions of people have received split and subunit vaccines, these vaccines are less effective in young children [6], [9], [11], [12], [13], [14]. LAIVs are made by combining the HA and NA genes of the target strain into an influenza virus genome that has been attenuated by adaptation to growth in colder temperatures [15], [16], [17], [18], [19]. Cold-adapted viruses are developed by continual passage of wild-type influenza virus in primary chick kidney cells [16], [20], [21]. LAIVs express the antigenic phenotype of the target strain with the attenuated phenotype of the cold-adapted backbone, greatly reducing replication at body temperatures [9], [21]. Replication of LAIV in the upper respiratory tract confers mucosal immunity and a circulating neutralizing antibody response.

Moreover, a cell-mediated immune response similar to infection with wild influenza, may offer broader protection against strain variants [9]. Flumist^®^ (MedImmune Vaccines Inc., Gaithersburg, Maryland, United States), a licensed LAIV, is delivered intranasally and is approved for use in healthy, non-pregnant subjects 2–49 years old [9], [19], [22], [23]. Its advantages over IIVs include simpler manufacturing, higher yield, faster release, and ease of use [22], [24]. Although no clear correlates of immunity for LAIV are defined, immune protection has been associated with serum hemagglutination-inhibition antibody and secretory immunoglobulin A (IgA) [25]. Also, randomized control trials found that LAIVs are more efficacious than IIVs in children, making them attractive for seasonal prevention efforts [25], [26], [27], [28], [29], [30]. FluMist^®^ contains the same antigens as the IIV for that season and has reported stability of 18 weeks at 2–8 °C [23]. LAIVs could be more widely used if they were stable at 2–8 °C for 1 year, or an entire influenza season.

To increase global vaccine supplies and support development of influenza vaccines in developing countries, the World Health Organization selected three developing-country manufacturers for a technology transfer initiative to strengthen capacity to produce LAIV against seasonal and pandemic influenza. This support granted manufacturers access to strains necessary for producing vaccines, especially the live-attenuated master donor virus strain from the Institute of Experimental Medicine (IEM; St. Petersburg, Russia) [24], [31]. Serum Institute of India Ltd. (SIIL) was selected to produce monovalent LAIV and trivalent seasonal LAIV using the IEM vaccine donor virus backbone for influenza A and B viruses [24], [32]. Although SIIL developed a commercial LAIV H1N1 vaccine, to achieve a one-year shelf life at 2–8 °C, the lyophilized product is costly and requires multiple handling steps before administration, limiting its programmatic suitability [22], [33].

The development of a low-cost, liquid formulation that is stable for an entire influenza season would contribute to greater acceptance by manufacturers and wider use of LAIV while decreasing production and distribution costs by removing the need for lyophilization [22].

Liquid LAIV formulation development at PATH included four steps: (1) screening potential excipients, (2) optimizing lead formulations, (3) validating lead formulations with an alternate influenza virus strain, and (4) confirming formulation stability with a higher titer of virus, agitation, and freeze–thaw exposure. This process produced a liquid LAIV formulation stable for 1 year at 2–8 °C.

## 2 Materials and methods

### 2.1 Virus potency assay

A tissue culture infectious dose_50_ (TCID_50_) procedure based on published methods was adapted for use with a colorimetric dye to determine the viral titer of influenza vaccine formulations [34], [35], [36]. A Madin-Darby canine kidney (MDCK) cell line from Influenza Reagent Resource (IRR) was selected for use in this assay (IRR FR-58, Lot 58851661). In addition, the reagent PrestoBlue (Life Technologies A13262) was selected to determine cell viability after infection. Cells were plated in 96-well plates (Nunc 167314) by adding 0.2 mL of 0.75 × 10^5^ cells/mL to each well and incubated at 37 °C with 5% CO_2_ for 18–24 h. Plated cells were washed and maintained in serum-free Eagle's minimum essential medium (ATCC) containing 10 mg/mL tosyl-phenylalanyl-chlorophenyl ketone (TPCK)-treated trypsin (cat# TRTVMF, Worthington Biochemical Corporation). Influenza virus was serially 10-fold diluted and MDCK cell monolayers were infected with influenza virus at dilutions of 10^−2^ to 10^−8^, with a total of six replicates per dilution and 0.2 mL inoculum per well. Plates were incubated for 6 days at 33 °C with 5% CO_2._ After viral replication, dye was added to detect cell viability. Assay performance was monitored by including a sample of LAIV (NASOVAC^®^) with each assay. Lyophilized LAIV (NASOVAC^®^) supplied by SIIL was stored at −30 °C (to prevent any measureable loss in titer during these studies) in single-use aliquots for an assay control. Titers were determined using the Reed-Muench formula [36]. The assay variability was ±0.23 log_10_ TCID_50_/mL. Live virus titers determined by the TCID_50_ assay were compared to those determined with the 50% Egg Infectious Dose (EID_50_) assay (the SIIL potency release assay) and found to be approximately 0.5 log_10_ lower. As the detection limit of the TCID_50_ assay is 2 × 10^3^ log_10_ TCID_50_/mL, changes in titer of greater than 0.5 log_10_ loss are detectable by this assay. LAIV formulation stability was defined as the time to 1 log_10_ loss of virus titer. Because this was a feasibility study, a 1 log loss threshold was selected, representing approximately 0.23 log_10_ TCID_50_/mL (variability of the TCID_50_ assay) plus a true loss of approximately 0.7 log_10_ TCID_50_/mL, giving approximately 1 log_10_ loss of initial virus titer.

### 2.2 Vaccine formulation

The starting materials, monovalent bulk influenza vaccines H1N1 (A/California/07/2009) and type B (B/Brisbane/60/2008), were provided by SIIL in sucrose phosphate glutamate (SPG) buffer at a concentration of 2 × 10^7^ log_10_ TCID_50_/mL. Formulations were prepared as indicated in Table 1. Formulations were monitored for pH, osmolality, and appearance by visual inspection. All formulations were within a pH range of 6.7–7.2 and an osmolarity range of 309–522 mOsm. The following excipients were evaluated: sucrose (Macron, cat#7723-04), glutamate (Sigma, cat#49621), sorbitol (Spectrum, cat#S0219), gelatin (Sigma–Aldrich, cat#G0262), bovine serum albumin (BSA) (Roche, cat#03117332001), arginine HCl (Sigma, cat#A4599), glycine (JT Baker, cat#0581-01), potassium phosphate monobasic (Macron, cat#7746-04), potassium phosphate dibasic (Sigma, cat#P3786), lactalbumin (Spectrum, cat#L3065), hydroxypropyl methylcellulose (HPMC) (Sigma, cat#423238), polyvinylpyrrolidone (PVP) (BASF Kollidon 17PF), recombinant human serum albumin (rHSA) (Novozymes), and human serum albumin (HSA) (MP Biomedicals, LLC, cat# 0882351). All excipients were United States Pharmacopeia (USP) grade reagents or Generally Regarded as Safe (GRAS). Formulations were prepared by diluting bulk vaccine into the formulations at a 1:10 ratio to achieve a final live virus titer of 2 × 10^6^ log_10_ TCID_50_/mL or where noted at a 1:5 ratio to achieve a final live virus titer of 1 × 10^7^ log_10_ TCID_50_/mL. All formulations were held in glass vials (West Pharmaceutical Services, Exton, PA) containing 1 mL for stability testing at 2–8 °C, 25 °C, and 33 °C. Loss was determined by TCID_50_ assay. Initially, we performed accelerated stability temperature testing at 37 °C, but most formulations lost greater than 1 log_10_ within 3 days. We selected a temperature of 33 °C for stability testing to improve resolution of the decay rate. Formulations were tested as indicated in each experiment or until 1 log_10_ loss was observed. Each experiment involved triplicate testing of one vial of each formulation at each time point. Lead formulations were repeated in subsequent formulation experiments. Stability during agitation was evaluated by horizontally shaking vials containing formulations for 24 h at 200 rpm at 20–25 °C. Freeze–thaw was performed by freezing vials at −20 °C for 2 h and then thawing at 20–25 °C for 1 h, for three cycles.

### 2.3 Statistical analysis

The decay rate was determined using the slope of the stability data in a linear regression model (GraphPad Prism software version 6). Data points at or below the limit of detection were excluded from the analysis. Statistically significant differences in the stability profiles of the lead excipients were evaluated by comparing slopes of linear regression lines using Prism software. A *p*-value <0.05 was considered significant. Final decay rates are from the lower 95% confidence interval based on three formulation experiments. Because this was an initial feasibility experiment, only one vial of each formulation was tested in triplicate in each experiment, limiting the performed statistical analyses.

## 3 Results

**Fig. 1:**
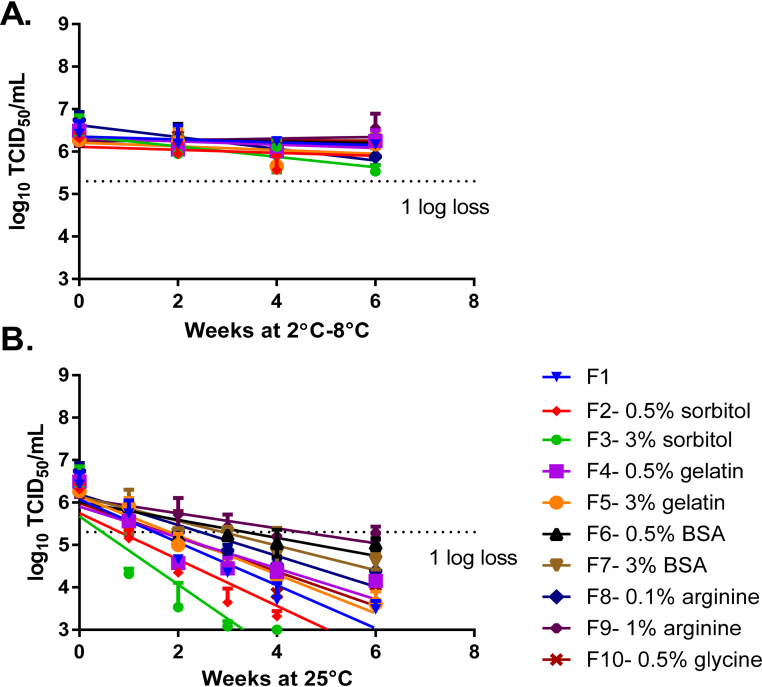
Screening of excipients. Formulations were prepared with LAIV H1N1 (A/California/07/2009) in SPG buffer and additional excipients were prepared at a titer of 2 × 10^6^ log_10_ TCID_50_/mL. The formulations were stored at 2–8 °C (A) and 25 °C (B), and their titer was measured by TCID_50_. Formulations were tested for up to 6 weeks or until a titer loss greater than 1 log was observed. *N* = 1 for each formulation tested by TCID_50_ in triplicate. Error bars represent the standard deviation of three TCID_50_ replicates. *Abbreviations*: BSA; bovine serum albumin.

In the first formulation stage, excipients were screened for their ability to improve the stability of LAIV compared to the vaccine in SPG buffer alone, as assessed by TCID_50_ (Fig. 1). All formulations were stable at 2–8 °C for 6 weeks (Fig. 1A). The addition of 1% arginine (Formulation 9) improved the stability of LAIV compared to SPG buffer alone (Formulation 1) at 25 °C. Sorbitol was the worst-performing excipient at 25 °C for both concentrations tested (Fig. 1B). Arginine and BSA were selected as the best-performing excipients for further evaluation.

**Fig. 2:**
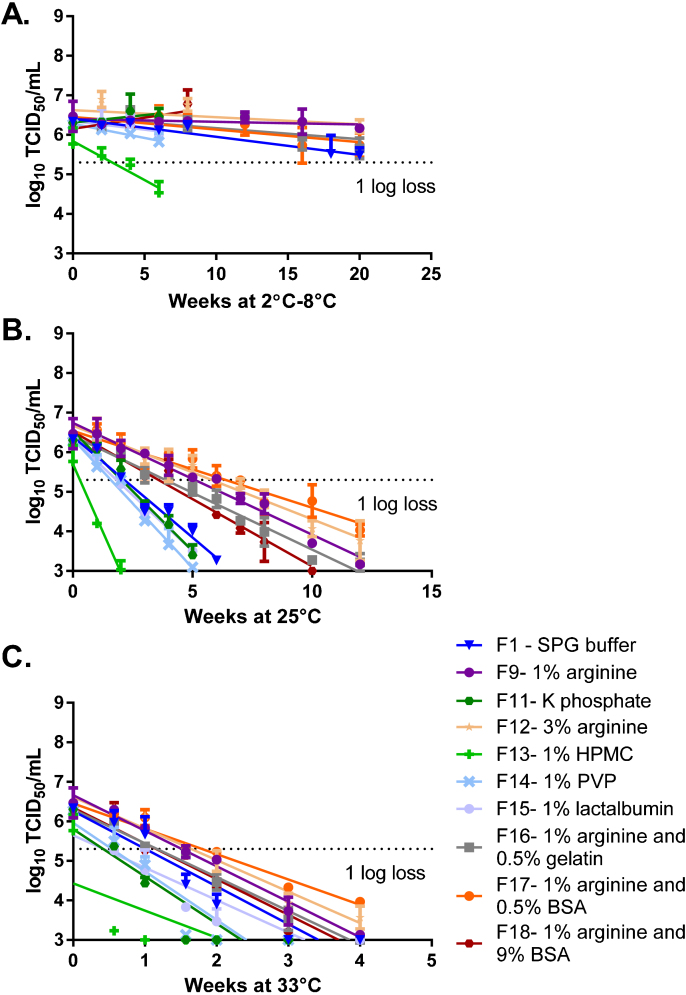
Formulation optimization. Formulations of LAIV H1N1 (A/California/07/2009) in SPG buffer and additional excipients were prepared at a titer of 2 × 10^6^ log_10_ TCID_50_/mL. The formulations were stored at at 2 °C–8 °C (A), 25 °C (B), and 33 °C (C), and their titer was measured by TCID_50_. Formulations were tested for up to 20 weeks or until a titer loss greater than 1 log was observed. N = 1 for each formulation tested by TCID_50_ in triplicate. Error bars represent the standard deviation of three TCID_50_ replicates. *Abbreviations*: SPG, sucrose phosphate glutamate; BSA, bovine serum albumin; HPMC, hydroxypropyl methylcellulose; PVP, polyvinylpyrrolidone.

In the second stage, lead formulations were optimized by combining excipients to further improve stability (Fig. 2). In addition, we tested wider concentration ranges for excipients to find the upper and lower limits of stability improvement. We also evaluated additional excipients: lactalbumin, HPMC, and PVP. As in the screening phase, arginine (Formulation-09) increased the stability of LAIV compared to SPG buffer alone (Formulation-01). Formulations containing arginine alone (Formulation-09 at 1% and Formulation-12 at 3%), 1% arginine with BSA (Formulation-17 at 0.5% BSA and Formulation-18 at 9% BSA), and 1% arginine with 0.5% gelatin (Formulation-16) showed stability for the length of the experiment (20 weeks) at 2–8 °C (Fig. 2A). Formulations containing 1% arginine held at 25 °C and 33 °C showed improved stability compared to those using SPG buffer alone. Formulations containing 3% arginine held at 25 °C and 33 °C showed an improvement in stability similar to formulations containing 1% arginine when compared to SPG buffer alone. The formulation containing 1% arginine and 0.5% BSA showed the largest stability improvement at 25 °C and 33 °C (Fig. 2B and C). A formulation containing 0.5% gelatin and 1% arginine showed stability similar to formulations of 1% arginine alone at 25 °C and 33 °C (Fig. 2B and C). HPMC and PVP appeared to destabilize LAIV and were not included in future formulations.

**Fig. 3:**
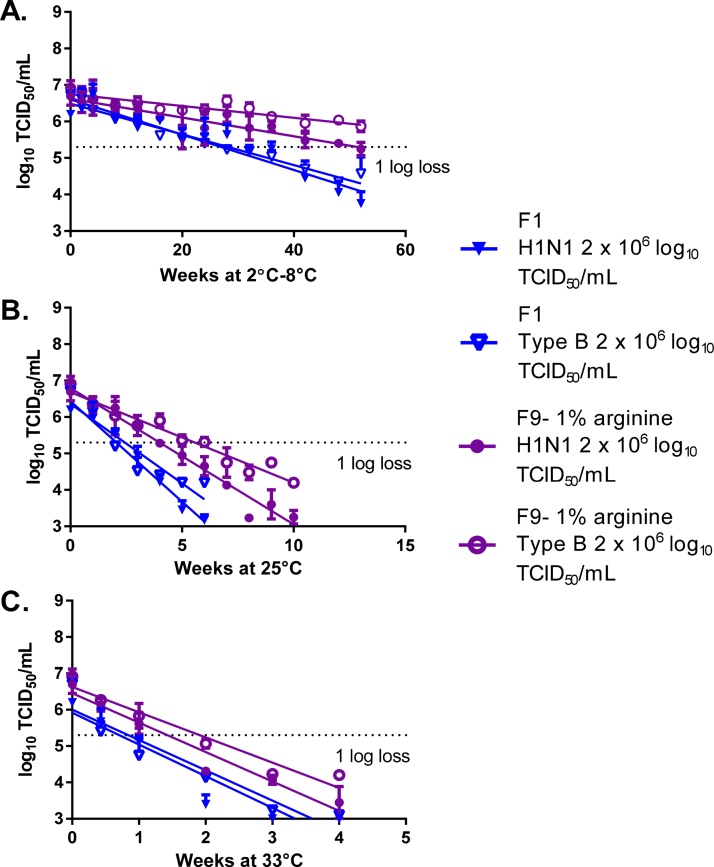
Formulation validation. Lead formulations were evaluated with an alternate LAIV strain, B/Brisbane/60/2008. Formulations of LAIV H1N1 or type B were prepared in SPG buffer or SPG buffer with 1% arginine at a titer of 2 × 10^6^ log_10_ TCID_50_/mL. The formulations were stored at 2–8 °C (A), 25 °C (B), and 33 °C (C), and their titer was measured by TCID_50_. Formulations were tested for up to 52 weeks or until a titer loss greater than 1 log was observed. *N* = 1 for each formulation tested by TCID_50_ in triplicate. Error bars represent the standard deviation of three TCID_50_ replicates.

During the validation stage, LAIV type B (B/Brisbane/60/2008) was formulated with the lead formulation containing 1% arginine (Formulation-09), and the use of SPG buffer alone (Formulation-01) was evaluated for comparison. LAIV formulations with 1% arginine with H1N1 were included to act as a bridge to previous formulation experiments (influenza titer of 2 × 10^6^ log_10_ TCID_50_/mL). Consistent with previous experiments, the use of 1% arginine led to statistically significant improvement in stability compared to use of SPG buffer alone at 2–8 °C (*p*-value <0.0001), 25 °C (*p*-value <0.0001), and 33 °C (*p*-value of 0.0002 and 0.003, respectively) for both H1N1 and type B (Fig. 3A–C).

**Fig. 4:**
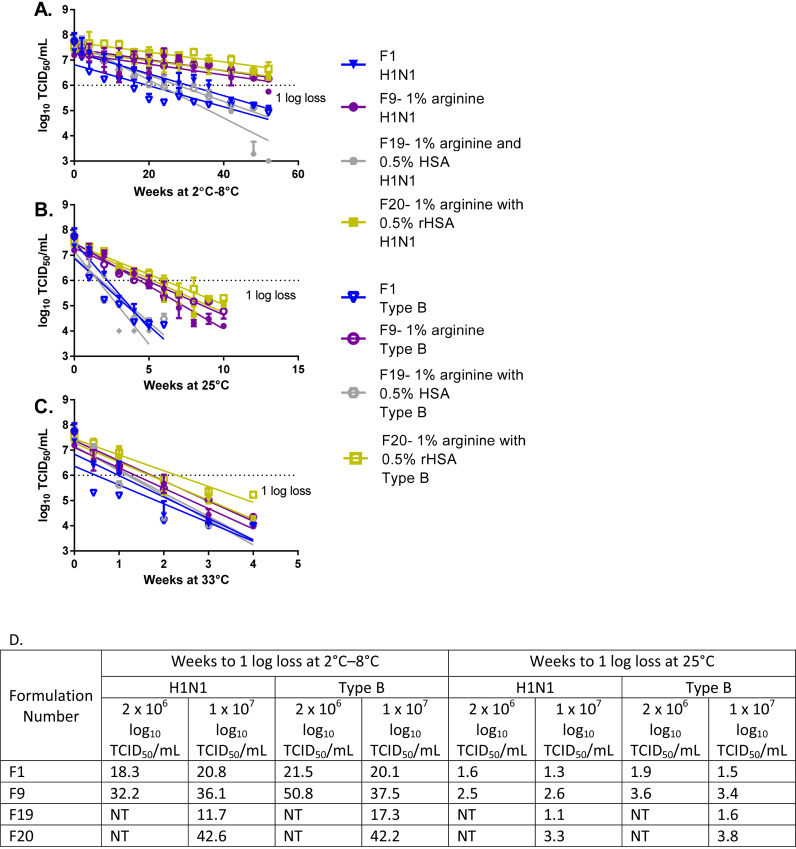
Confirmation of lead formulations containing increased LAIV titer. Formulations of LAIV H1N1 (A/California/07/2009) or type B (B/Brisbane/60/2008) in SPG buffer and selected excipients were prepared at a titer of 1 × 10^7^ log_10_ TCID_50_/mL. The formulations were stored at at 2–8 °C (A), 25 °C (B), and 33 °C (C), and their titer was measured by TCID_50_. Formulations were tested for up to 52 weeks or until a titer loss greater than 1 log was observed. *N* = 1 for each formulation tested by TCID_50_ in triplicate. Error bars represent the standard deviation of three TCID_50_ replicates. (D) Summary of time (in weeks) to 1 log loss for the lead formulations identified represents the lower 95% confidence interval from the average of all three experiments lead formulations were evaluated. NT = not tested.

To confirm the formulations selected, we tested a higher concentration (1 × 10^7^ log_10_ TCID_50_/mL) of LAIV strains (H1N1 and type B) (Fig. 4). The higher titer better mimics what would be present in a vaccine product. In addition, to avoid using animal-derived raw materials, we tested formulations that substituted HSA or rHSA for BSA. Formulations containing 1% arginine (Formulation-09) and 1% arginine with 0.5% rHSA (Formulation-20) with both H1N1 and type B were stable for the length of the experiment (42.6 weeks H1N1 and 42.2 weeks type B using the lower 95% confidence Interval) at 2–8 °C. H1N1 formulations with 1% arginine improved stability compared to formulations with SPG buffer alone (*p*-value <0.0001), but not as much as formulations containing 1% arginine with 0.5% rHSA (Formulation-20, *p*-value <0.0001) at 25 °C. However, at 33 °C, H1N1 formulations with 1% arginine or 1% arginine with 0.5% rHSA showed similar stability. Type B formulations with 1% arginine showed statistically significant improvements in stability compared to formulations with SPG buffer alone at 2–8 °C and 25 °C (*p*-values <0.0001). Type B formulations with 1% arginine and 0.5% rHSA showed slightly improved stability compared to 1% arginine alone at 25 °C and 33 °C. The addition of 0.5% rHSA appeared to slightly improve the stability of formulations for both LAIV H1N1 and type B strains compared to stability with 1% arginine alone, although these differences were not statistically significant.

By using the decay rate, we calculated the number of weeks to reach 1 log_10_ loss at 2–8 °C or 25 °C using the lower bound of the 95% confidence interval (Fig. 4D). Based on three stability experiments, for H1N1 formulated at a titer of 2 × 10^6^ log_10_ TCID_50_/mL with SPG and 1% arginine, the lower 95% confidence interval for the time to reach 1 log loss was 32.2 weeks and 2.5 weeks at 2–8 °C and 25 °C, respectively.

One experiment was performed with type B formulated at a titer of 2 × 10^6^ log_10_ TCID_50_/mL with SPG and 1% arginine. The lower 95% confidence interval for the time to 1 log_10_ loss at 2–8 °C and 25 °C was 50.8 and 3.6 weeks, respectively. Stability testing with a higher dose of LAIV was performed only once, but the time to 1 log_10_ loss for H1N1 at a titer of 1 × 10^7^ log_10_ TCID_50_/mL formulated in SPG with 1% arginine was 36.1 weeks and 2.6 weeks at 2–8 °C and 25 °C, respectively. For Type B, the time to 1 log_10_ loss at the higher concentration was 37.5 weeks and 3.4 weeks. Formulations with SPG, 1% arginine, and 0.5% rHSA at a titer of 1 × 10^7^ log_10_ TCID_50_/mL showed a time to 1 log_10_ loss of 42.6 weeks and 3.3 weeks for H1N1 and 42.2 weeks and 3.8 weeks for type B at 2–8 °C and 25 °C, respectively.

**Fig. 5:**
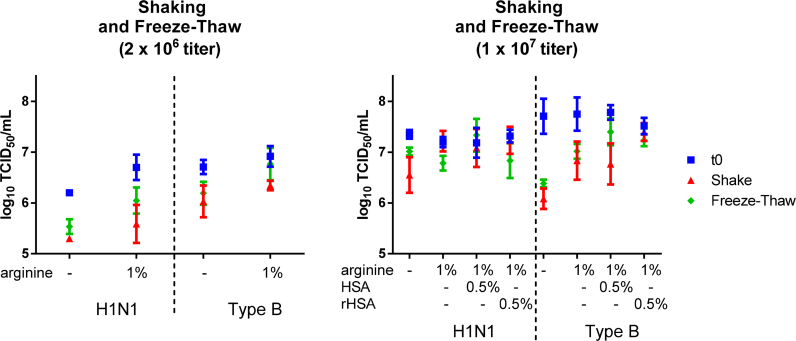
Effect of agitation and freeze–thaw stresses on LAIV titer. Formulations of LAIV H1N1 or Type B in SPG buffer and selected excipients were prepared at a titer of 2 × 10^6^ log_10_ and 1 × 10^7^ log_10_ TCID_50_/mL. Agitation was performed by shaking vials horizontally for 24 h at 200 rpm at ambient temperature (20–25 °C). Freeze–thaw was performed by freezing vials at −20 °C for 2 h, then thawing at 20–25 °C for 1 h for a total of 3 freeze–thaw cycles. After the stress tests were completed, the formulations were tested by TCID_50._ Error bars represent standard deviation of 3 formulation vials tested in triplicate.

We also assessed the effects of agitation and freeze–thaw to confirm stability. Formulations were prepared with LAIV strains at titers of 2 × 10^6^ and 1 × 10^7^ log_10_ TCID_50_/mL. Agitation and freeze–thaw were performed to mimic what a vaccine vial might encounter during shipment or storage (Fig. 5). The addition of rHSA to formulations of H1N1 and type B LAIV at titers of 1 × 10^7^ log_10_ TCID_50_/mL improved stability during freezing and agitation compared to formulations with 1% arginine alone and SPG buffer alone.

## 4 Discussion

This work identified a stable liquid LAIV formulation for potential use in monovalent, trivalent, or quadrivalent seasonal influenza vaccines. Through several formulation stages and testing by TCID_50_, we identified a formulation with stability of 42 weeks at 2–8 °C for type A and type B influenza LAIV. This formulation consists of SPG with 1% arginine and 0.5% rHSA.

Formulation stability of LAIV is influenced by several factors (e.g., strain, pH buffer, aggregation) [37], [38]. SPG is a commonly used stabilization buffer and is present in several widely used vaccines, such as measles, mumps, rubella (MMR) vaccine and FluMist^®^ [23], [39]. Although sorbitol is a well-established osmolyte for stabilizing proteins and preventing aggregation [37], [40], [41], it did not stabilize the LAIV strains tested in this study.

Protein-based excipients are commonly used as stabilizers. Because of their size and surface activity, they accumulate at the air-liquid interface, potentially shielding formulation components from surface tension [37], [42]. Amino acids use various mechanisms to stabilize formulations and are used with a variety of biomolecules [37], [43], [44], [45]. l-Arginine and l-glycine, for example, are common excipients for pharmaceutical applications [40], [43], [46], [47]. l-Arginine improves stability in a range of applications, including antibodies, vaccine antigens, and fusion proteins [43], [46], [48], [49]. l-Arginine may aid protein refolding, solubilization, the prevention of aggregation, and the prevention of nonspecific adsorption [43]. In this study, l-arginine at 1% greatly increased the stability of the LAIV strains tested.

The polymers PVP and HPMC were included because of their use in mucosal formulations and their potential stabilizing benefits [50]. Because formulations containing these polymers were less stable than formulations with buffer alone, they were removed from the final formulation experiments. Future work needs to evaluate the potential benefits of adding these polymers to the stable formulation identified in this work.

In our experiments, only l-arginine with rHSA increased the stability of the LAIV strains tested. rHSA provided a minor improvement over formulations containing arginine alone—an improvement observed most dramatically during freeze–thaw and agitation experiments with type B. If a vaccine can withstand a freeze–thaw cycle, freezing can be used to further increase shelf life by keeping the vaccine frozen at the manufacturing site and then initiating storage at 2–8 °C upon shipment or during short-term storage at the clinical site. Although BSA showed improved stability in the initial stages, we removed animal-derived products from our formulations and substituted rHSA because animal-derived reagents may cause faith-based concerns or concerns about possible contamination with endogenous diseases. In addition, HSA formulations showed increased turbidity over time, which may have decreased stability. The turbidity could be due to the fatty acid content in HSA that is not present in rHSA. HSA formulations could be revisited with a fatty-acid HSA reagent in future experiments.

In conclusion, this paper describes the successful identification of stable liquid LAIV formulations for use in seasonal vaccination programs. A liquid LAIV formulation allows for a vaccine that is easier to use, potentially leading to greater acceptance and wider adoption of seasonal vaccination. The formulations identified in this work stabilized LAIV strains H1N1 (A/California/07/2009) and B (B/Brisbane/60/2008) for approximately one year (42 weeks using lower 95% confidence interval) at 2–8 °C and for up to approximately 4 weeks at 25 °C. FluMist^®^ was developed for vaccine strains based on use of the A/Ann Arbor/6/60 or B/Ann Arbor/6/66 genetic background. The role of viral genetic differences in formulation stability has not been investigated. Modern seasonal influenza vaccines contain three or four strains, so a formulation must sufficiently stabilize all included strains. Although we had access to only two seasonal strains for our studies, the results demonstrate that both could be stabilized for an entire influenza season. Ongoing work with a manufacturer is evaluating the addition of 1 log of virus to the vaccine formulation.

## Conflict of interest

The authors are employed by PATH, a nonprofit international health organization, and received grant funding to advance research to assess the technical and commercial viability of improving the stability of vaccines of importance to developing countries.

## Figures and Tables

**Table 1:** LAIV liquid formulations evaluated.

Formulation number	Components	Influenza strain tested
F1	SPG buffer	H1N1 and type B
F2	SPG + 0.5% sorbitol	H1N1
F3	SPG + 3% sorbitol	H1N1
F4	SPG + 0.5% gelatin	H1N1
F5	SPG + 3% gelatin	H1N1
F6	SPG + 0.5% BSA	H1N1
F7	SPG + 3% BSA	H1N1
F8	SPG + 0.1% arginine	H1N1
F9	SPG + 1% arginine	H1N1 and type B
F10	SPG + 0.5% glycine	H1N1
F11	SPG 100 mM potassium phosphate	H1N1
F12	SPG + 3% arginine	H1N1
F13	SPG + 1% HPMC	H1N1
F14	SPG + 1% PVP	H1N1
F15	SPG + 1% lactalbumin	H1N1
F16	SPG + 1% arginine and 0.5% gelatin	H1N1
F17	SPG + 1% arginine and 0.5% BSA	H1N1
F18	SPG + 1% arginine and 9% BSA	H1N1
F19	SPG + 1% arginine and 0.5% HSA	H1N1 and type B
F20	SPG + 1% arginine and 0.5% rHSA	H1N1 and type B

*Abbreviations*: SPG, sucrose phosphate glutamate; BSA, bovine serum albumin; HPMC, hydroxypropyl methylcellulose; PVP, polyvinylpyrrolidone; HSA, human serum albumin; rHSA, recombinant human serum albumin.
